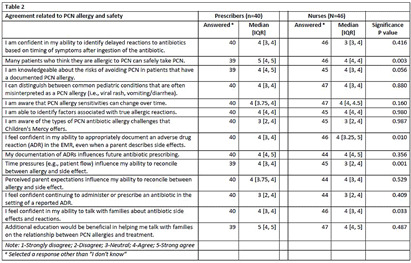# Perceptions on Penicillin Allergy Labels among Nurses and Prescribers in Three Pediatric Urgent Care Sites

**DOI:** 10.1017/ash.2024.158

**Published:** 2024-09-16

**Authors:** Elizabeth Monsees, Amanda Nedved, Sarah Suppes, Megan Whitt, Brian Lee, Diane Petrie, Adrienne Olney, Rana El Feghaly

**Affiliations:** Children’s Mercy Hospital, Performance Excellence; Children’s Mercy Kansas City; University of Missouri Kansas City School of Medicine; Children’s Mercy Hospital; Children’s Mercy Hospitals and Clinics

## Abstract

**Background:** National guidelines recommend penicillins (PCN) as first-line treatment for many common pediatric infections in the outpatient setting. Although less than 1% of the United States population has a true, IgE-mediated PCN-allergy, approximately 10% of patients are labeled with a PCN-allergy. Accurate adverse drug reaction (ADR) documentation plays an important role in this over-labeling. We have previously shown that nurses feel assessment and documentation of PCN-allergies are critical to their role. However, additional evidence purports nurse hesitancy to interrogate allergy accuracy or reclassify parent’s response to side effect. Our objective was to explore frontline clinicians’ confidence in assessing, documenting, and responding to PCN-allergy labels. **Methods:** To expose barriers and prioritize improvement ideas for a multidisciplinary quality improvement (QI) project aimed to improve PCN-allergy labeling in our pediatric urgent care clinics, we deployed this investigator-developed survey to prescribers and nurses. It’s comprised of 14-questions scored on a 5-point Likert scale (4 demographic, 4 PCN/safety, 3 allergy types, 4 allergy documentations, 3 treatment options), and 1 optional free-text. We used descriptive statistics to compare survey responses between prescribers and nurses and evaluated free text comments for themes. **Results:** Eighty-seven clinicians across 3 sites participated, with a response rate of 35%, with variation by sites (25.3% to 41.4%). Forty-one percent of (n=36) responders have been in practice >15 years and 40.2% (n=35) have worked at our hospital > 15 years (Table 1). Overall, perceived knowledge of PCN-allergies and safety was favorable (Table 2). Prescribers reported higher confidence with: 1) perceiving many patients who believe they are allergic to PCN can safety take PCN (prescribers median=5 [IQR: 4, 5] vs. nurses median=4 [4,4], p = 0.003); and 2) perceiving that time pressures influenced their ability to reconcile allergies and side effects (prescribers median=4 [4, 5] vs. nurses median=3 [2, 4], p = 0.001). Both prescribers and nurses reported lower confidence in continuing to administer or prescribe an antibiotic in the setting of a reported ADR. Thirteen respondents (15%) provided comments with specific requests for additional family education and practice guidance, including the referral process to subspecialty clinics for PCN-allergy testing. **Conclusions:** Our survey results identified barriers to accurate PCN-allergy labels, including knowledge on documentation, time pressures, hesitancy to challenge parent report, and uncertainty on referral process for PCN-allergy testing. This survey will inform future drivers for our QI. Opportunities include electronic medical record refinement, improving referrals to PCN-allergy de-labeling clinics, and the development of scripted education to guide family discussions.

**Disclosure:** Rana El Feghaly: Merck- grant funding. Amanda Nedved: Contracted Research – Merck

**Figure:**
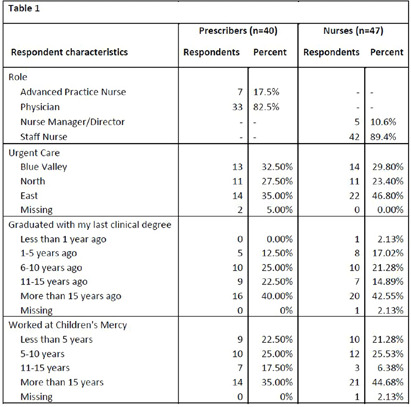

**Figure:**